# Supplementary figures and images for: Repertoire of Naturally Acquired Maternal Antibodies Transferred to Infants for Protection Against Shigellosis
Source: Front Immunol. 2021 Oct 15;12:725129. doi: 10.3389/fimmu.2021.725129 (PMC8554191; doi:10.3389/fimmu.2021.725129)

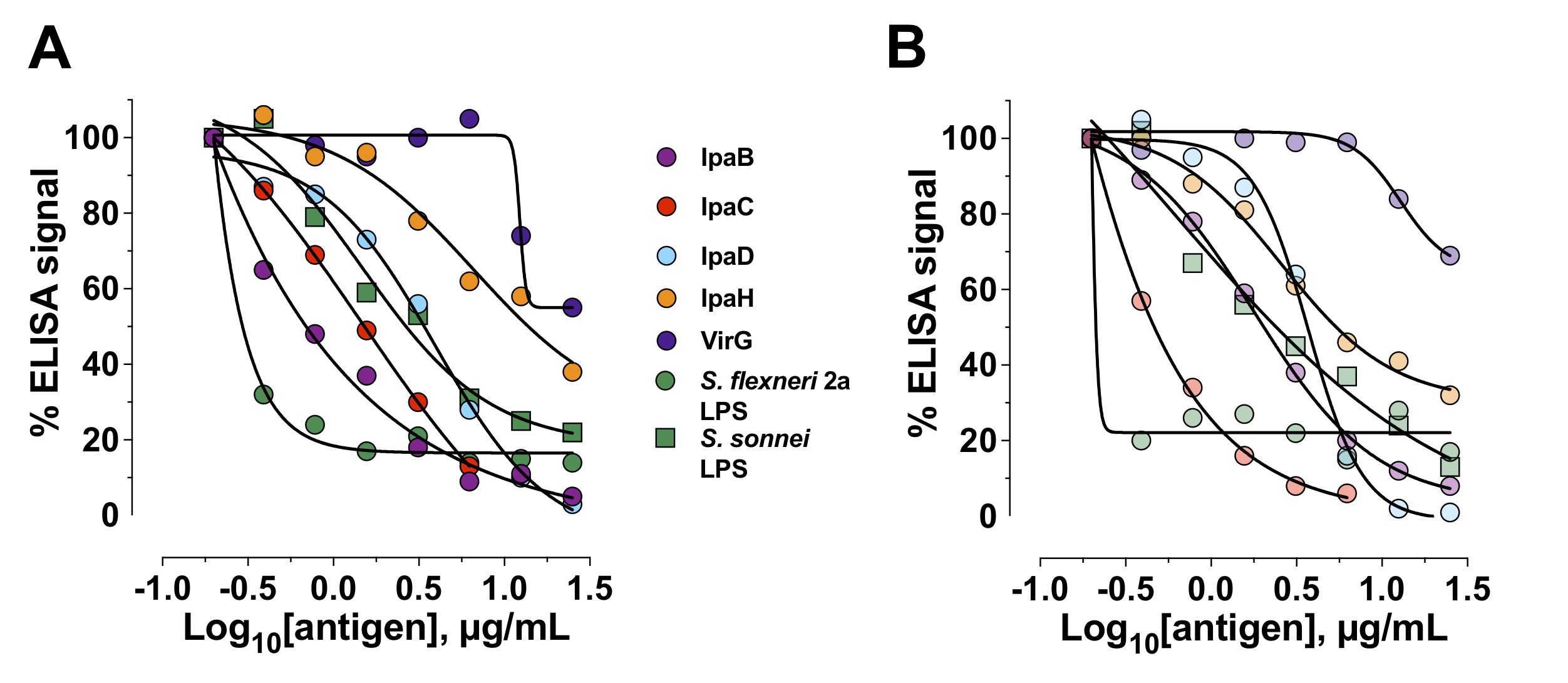

Supplement: Supplementary file 1 [file Image_1.tiff]

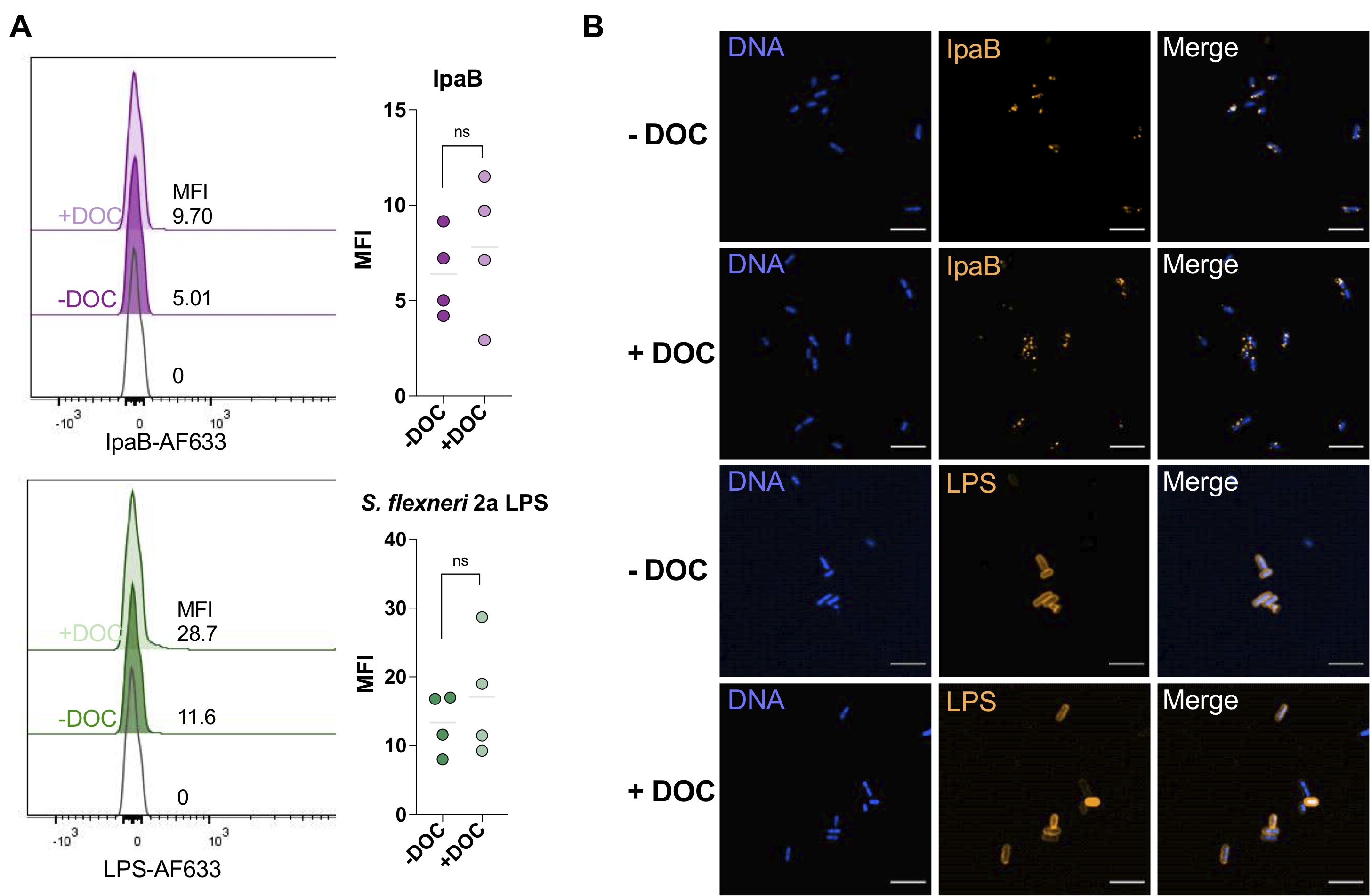

Supplement: Supplementary file 2 [file Image_2.tiff]
